# Commentary: Obesity and Weight Gain in Pregnancy and Postpartum: an Evidence Review of Lifestyle Interventions to Inform Maternal and Child Health Policies

**DOI:** 10.3389/fendo.2019.00163

**Published:** 2019-03-26

**Authors:** Helen Skouteris, Helena J. Teede, Shakila Thangaratinam, Cate Bailey, Jo-Anna Baxter, Heidi J. Bergmeier, Cheryce Harrison, Briony Hill, Brian Jack, Laura Jorgensen, Siew Lim, Thabo Matsaseng, Cynthia Montanaro, Eric Steegers, Judith Stephenson, Hildrun Sundseth, Ana Luiza Vilela Borges, Ruth Walker, Leanne Redman, Jacqueline Boyle

**Affiliations:** ^1^Monash Centre for Health Research and Implementation, School of Public Health and Preventive Medicine, Monash University and Monash Health, Clayton, VIC, Australia; ^2^Monash Partners Advanced Health Research Translation Centre, Clayton, VIC, Australia; ^3^Monash Health, Clayton, VIC, Australia; ^4^Women's Health Research Unit, Centre for Primary Care and Public Health, Barts and The London School of Medicine and Dentistry, Blizard Institute, London, United Kingdom; ^5^Centre for Global Child Health, The Hospital for Sick Children, Toronto, ON, Canada; ^6^Department of Nutritional Sciences, University of Toronto, Toronto, ON, Canada; ^7^Department of Family Medicine, Boston University School of Medicine, Boston, MA, United States; ^8^Katie's Team, East London, United Kingdom; ^9^UNDP-UNFPA-UNICEF-WHO-World Bank Special Programme of Research, Development and Research Training in Human Reproduction, Department of Reproductive Health and Research, World Health Organization, Geneva, Switzerland; ^10^Wellington-Dufferin-Guelph Public Health, Guelph, ON, Canada; ^11^Department of Obstetrics and Gynaecology, Erasmus Medical Centre – Sophia Children's Hospital, Rotterdam, Netherlands; ^12^Institute for Women's Health, University College, London, United Kingdom; ^13^European Institute of Women's Health, Dublin, Ireland; ^14^Public Health Nursing Department, University of São Paulo, São Paulo, Brazil; ^15^Reproductive Endocrinology and Women's Health Laboratory, Pennington Biomedical Research Center, Baton Rouge, LA, United States

**Keywords:** gestational weight gain, postpartum weight retention, obesity, preconception body mass index, healthy lifestyle intervention, pregnancy

We read with interest the recent review published in *Frontiers in Endocrinology* that was focused on obesity and weight gain in pregnancy and postpartum. The review of systematic reviews and meta-analyses, investigating the effects of lifestyle interventions on gestational weight gain (GWG) and postpartum weight retention (PPWR), provides evidence showing that lifestyle interventions can reduce excess weight gain and associated risk factors. We agree unconditionally that the burden of maternal and childhood obesity needs to be reduced urgently.

There is a clear policy mandate internationally to prevent maternal obesity given the adverse impact on maternal and child health, and the challenges of treating obesity, which are intensive, costly, largely ineffective, and unsustainable at a population level. The World Health Organization (1), National Institute for Health and Care Excellence (2), the Australian Medical Association (3), the Australian National Health and Medical Research Council Obesity Translation Committee (4) and the US Institute of Medicine (5), have unanimously called for targeted efforts to improve lifestyle behaviors during pregnancy to optimize gestational weight gain, prevent postpartum weight retention and improve short- and long-term maternal health and long-term child health outcomes.

In concordance with the findings of Farpour-Lambert et al. (6), we have reported lifestyle intervention during pregnancy reduces GWG, gestational diabetes, and cesarean births (7, 8), and in postpartum is effective for weight loss, weight gain prevention, and improving metabolic and reproductive outcomes (9, 10). With demonstrated efficacy and an extensive evidence base now established, the consolidation of current evidence and identification of specific gaps is underway, which is vital to inform further trials that address these gaps and advance, rather than simply expand, the field. Furthermore, effective low-intensity and low-cost lifestyle interventions in pregnancy and postpartum need to be implemented at scale to prevent excessive weight gain and to promote healthy lifestyle (11).

Whilst we agree with Farpour-Lambert et al. (6) that multicomponent lifestyle strategies should be offered to women in pregnancy and postpartum, we emphasize that the preconception period is just as important and cannot be ignored in systems level approaches to preventing maternal and childhood obesity (12, 13). It is imperative to improve women's health status before pregnancy. A high body mass index (BMI) in the preconception period reduces fertility and increases complications when pregnancy does occur including gestational diabetes, gestational hypertension, preeclampsia, early pregnancy loss, congenital fetal anomalies, large-for-gestational-age infants, preterm birth and still birth (14) as well as cesarean section (15, 16) and newborn morbidity from shoulder dystocia (17) and increases the risk of subsequent offspring obesity (18). Clear preconception health promotion priorities, related to healthy diet, weight management, dietary supplementation, physical activity, substance use and more, await implementation given the intergenerational effects of sub-optimal lifestyle behaviors from conception.

As a direct response to the growing prevalence of overweight and obesity among women before, during and after pregnancy, a team of Australian researchers formed the Health in Preconception, Pregnancy and Post Birth (HiPPP) Collaborative in 2013 (13). HiPPP encompassed multidisciplinary expertise and engaged stakeholders across community, government, private and public health services, workplaces, primary care, and consumer advocates/patient representatives. HiPPP is a network with the primary aim of improving lifestyle and preventing maternal obesity. The HiPPP network is strengthened by partnership, research, capacity building, knowledge translation and collaboration.

Despite the vast body of research to date, there is currently no international consensus on guidelines around preconception, pregnancy and postpartum healthy behaviors and prevention of weight gain, with only 42% addressing preconception and 13% addressing postpartum phases (19). Furthermore, no country has implemented systems level practice and policy evidence-based strategies targeting preconception, pregnancy and postpartum life stages to prevent obesity. The HiPPP network is focused on addressing the few clear remaining gaps in evidence around efficacy, yet primarily we are progressing implementation research and translation of existing evidence into policy and practice. In this context, we are seeking to collaborate on areas including innovative and generationally relevant electronic health strategies (20, 21). Evidence synthesis, guideline development, strategic prioritized implementation research, translation, capacity building and collaboration are now crucial to drive evidence into practice, improve lifestyles, reduce the obesity epidemic and deliver health impact for the benefit of today's and future generations. We are responding to this need.

HiPPP has now expanded internationally. In 2018, as an alliance of global leaders in the area of preconception and pregnancy health, including early career researchers and consumer advocates, we (the authors on this paper) came together with a vision for improving the health of all women of reproductive age, expectant mothers and their children. Together we are focused on stakeholder and consumer engagement, evidence synthesis, guideline development, workforce capacity building, priority setting, implementation research, translation and scale up for health impact. We are developing a consumer and community involvement (CCI) framework to guide our HiPPP global alliance program of research and our evidence synthesis and guideline appraisal are well advanced.

There is a now a clear and imperative call to action to consolidate and advance current evidence into practice and policy; Farpour-Lambert et al.'s (6) findings support this call to action. Efficacy is established and now is an opportune time to pool existing study data internationally, explore core components, delivery methods, and implementation strategies to demonstrate broader effectiveness of lifestyle interventions in preconception, pregnancy, and postpartum. This includes implementation research, behavior change taxonomy and health economic analyses of value (cost and quality). These activities are well underway and ultimately will enable the international community to implement effective interventions at scale to reduce the prevalence of maternal obesity and improve related health outcomes for both women and future generations.

## Author Contributions

All authors were part of an inaugural HiPPP global alliance that met in Prato, Italy, 27–28th September, 2018. HS, HT, and JA-B led the writing of this paper and all other authors read drafts and provided comments.

### Conflict of Interest Statement

The authors declare that the research was conducted in the absence of any commercial or financial relationships that could be construed as a potential conflict of interest.
